# Design of Experiments-Based Fire Performance Optimization of Epoxy and Carbon-Fiber-Reinforced Epoxy Polymer Composites

**DOI:** 10.3390/polym15204096

**Published:** 2023-10-16

**Authors:** Christoph M. Pöhler, Marwa Hamza, Torsten Kolb, Erik V. Bachtiar, Libo Yan, Bohumil Kasal

**Affiliations:** 1 Centre for Light and Environmentally-Friendly Structures, Fraunhofer Institute for Wood Research Wilhelm-Klauditz-Institut WKI, 38108 Braunschweig, Germany; torsten.kolb@wki.fraunhofer.de (T.K.); bohumil.kasal@wki.fraunhofer.de (B.K.); 2 Independent Researcher, 38108 Braunschweig, Germany; marwa639@outlook.sa (M.H.); erik.valentine.bachtiar@gmail.com (E.V.B.); 3 Department of Organic and Wood-Based Construction Materials, Technische Universität Braunschweig, 38108 Braunschweig, Germany

**Keywords:** thermosetting resin, design of experiments, fire retardation, carbon fibers

## Abstract

The fire performance of epoxy and carbon-fiber-reinforced polymer (CFRP) composites with and without fire retardants (FR) (i.e., ammonium polyphosphate (APP), aluminum trihydroxide (ATH), melamine (MEL), expandable graphite (EG)) was investigated. A design of experiment (DoE) approach was applied to study the single- and multifactorial effects of FR. The fire performance of epoxy and CFRP was evaluated by limiting the oxygen index (LOI) and heat release, which were obtained by limiting the oxygen index test and cone calorimetry. It was found that mixtures of 70 wt.-% epoxy, 24.6 wt.-% of APP, and 5.4 wt.-% MEL resulted in the highest LOI level of 45 within tested groups for epoxy resin and also for CFRP specimens (LOI level of 39). This mixture also resulted in the lowest average heat release rate (HRR_180s_) of 104 kW·m^−2^ and a spec. total heat release (THR_600s_) of 1.14 MJ·m^−2^·g^−1^, indicating the importance of balancing spumific and charring agents in intumescent systems and synergy thereof.

## 1. Introduction

Fiber-reinforced polymer (FRP) composites constitute a lightweight material class with outstanding specific strength and stiffness. With high performance at low weight, FRP has been widely influencing today’s engineering solutions [1]. However, due to the limited glass transition temperatures being typically in the range of 50–200 °C and general inflammability of epoxy polymers, the use of FRP in a load-bearing structure with high fire-resistance is still scarce. National standards and safety regulations, e.g., EN 13501-1, EN 4102-1, for building constructions, require fire retardancy and structural integrity in events of fire of up to 30, 60, or 90 min depending on the material classification [2,3,4].

By intervening at different stages of the fire process, such as at the ignition stage, the flame spread and rapid-fire development can be hindered. During pyrolysis, the polymer in the condensed phase exposed to heat releases combustibles into the gas phase. In the presence of oxygen, these combustibles are oxidized exothermally, transmitting heat back to, inter alia (i.a.), the condensed phase and thus, contributing to further pyrolysis. FRP composites based on epoxy resins are inherently inflammable. Aliphatic parts in their matrix make them susceptible to burning and rapid-fire development. Possible ways to increase the fire resistance for FRP-reinforced structural elements are intumescent coatings and external thermal insulation [5,6,7,8]. Another approach, derived from the development of fire-retardant thermoplastics, is to incorporate fire retardants into the polymeric matrix system, either by the dispersion of fire retardants into the mixture of resin and hardener, as co-reactants or by introducing certain functional groups into the polymer [9,10,11]. Fire retardation works, e.g., by the dilution of the gas phase, deprivation of energy, shielding and insulating the condensed phase by carbonaceous char formation, foaming, or a combination of these [12]. As temperatures increase, any polymer will undergo a structural change, from a glassy to rubbery state, and lose mechanical integrity. Not only flammability but also thermal resistance is significant in the event of fire. Hence, for FRPs as a lightweight alternative to conventional construction and retrofitting materials, fire and thermal performances are major issues to be solved [13]. Cost-effectiveness, health, and environmental concerns are some of the main factors that drive research incessantly in the field of optimization [14].

Although research and literature on the fire retardants considered in this study are ample, the effects of incorporating those fire retardants directly in the matrix of a fiber-reinforced polymer have been rarely illuminated. This research aims to provide insight on the synergistic and antagonistic effects of incorporated mixtures of fire retardants based on a statistical approach. A design of experiments was carried out on several types of fire retardants to optimize the fire retardancy of epoxy resins and carbon fiber-reinforced polymers. The parameters for screening different fire retardants were their miscibility and effects on the working life, meaning the time a mixed epoxy system is still within the limits of viscosity and temperature to be used for application. For screening, aluminum hydroxide (ATH), ammonium polyphosphate (APP), melamine (MEL), pentaerythritol (PER), and expandable graphite (EG) were chosen due to their promising fire-retardant behavior, low toxicity, and different fire retardation mechanisms [12,15,16].

Subsequently, the flame retardants that had passed the screening were selected and a statistical test plan was developed based on practical limitations, e.g., effects on viscosity and curing. Mixtures of epoxy and fire retardants were produced and examined in the Limiting oxygen index (LOI) test, cone calorimetry test, and thermo-gravimetric analysis (TGA). A regression model was calculated to find the optimum mixture regarding fire retardancy within the considered test space. Additionally, mixtures with limiting oxygen index (LOI) levels ranked 30 or above were investigated as fire retardant matrix systems for CFRP, in cone calorimetry and tensile testing. Lower-ranking mixtures were excluded from further investigation due to their limited fire-retardant effect [17]. Finally, the calculated optimum from the regression model was tested by additional verification mixtures and results from the fire tests that were compared and discussed.

## 2. Materials and Methods

### 2.1. Materials

#### 2.1.1. Epoxy

The epoxy matrix used in this work is a two-component system of a bisphenol A-based epoxy-resin (Ampreg 31) and amine-based hardener (Ampreg 3X Standard Hardener) with a mixture ratio of 100:26 from Gurit GmbH, Zurich, Switzerland. The resulting mixed viscosity at 25 °C amounts to 1000 cP with a density of 1.08 g/cm^3^. The glass transition temperature T_g_ at ambient temperature curing is 59 °C.

#### 2.1.2. Carbon Fibers

Carbon fiber fabric from Zoltek Corporation, Bridgeton, MO, USA was acquired from R&G GmbH, Waldenbuch, Germany. The unidirectional fabric (UD200) consisted of 200 g/m^2^ Zoltek PX35 50K carbon fibers in the longitudinal direction and perpendicular glass fibers (34 dtex) connected by a polyester stitch (76 dtex) with a total areal density of 224 g/m^2^. The resulting fiber volume fraction of CFRP amounted to 49.3% with a coefficient of variation (CoV) of 11.1%. Details for the production of CFRP are discussed later, in Section 2.4.

#### 2.1.3. Fire Retardants

Aluminum hydroxide (ATH) with a relative content of AL_2_O_3_ of 64.9 wt.-% and a loss on ignition of 34.6 wt.-% was purchased as Micral^®^ 855 from J.M. Huber Finland OY, Hamina, Finland [18]. The density is 2.42 g/cm^3^, and the fineness as a median particle diameter (D50) is 2 µm. Ammonium polyphosphate (APP), [NH_4_PO_3_]_n_ with a chain length (n) greater than 1000 was obtained as Exolit^®^ AP 422 from Clariant GmbH, Munich, Germany. The density is 1.9 g/cm^3^ and the median particle size (D50) was specified as 17 µm [19]. Melamine (C_3_H_6_N_6_) with min. 99.8% purity was purchased as Melafine^®^ from OCI Nitrogen BV, Geleen, Netherlands [20]. The density was stated to be 1.57 g/cm^3^ with a particle size less than 40 µm. Pentaerythritol (C_5_H_12_O_4_) was obtained as Charmor™ PM15 from Perstorp AB, Malmö, Sweden. The density according to the data sheet is 1.4 g/cm^3^ and particle sizes are less than 15 µm (min. 98 wt.-%) [21]. Expandable graphites (EG) in two different finenesses are GHL PX 96/-1 with median particle size of 70–110 µm and an expansion rate of min. 100 mL/g and GHL PX 95N with a median particle size of 360–420 µm and an expansion capacity of min. 250 mL/g and were purchased from Georg H. Luh GmbH, Walluf, Germany [22,23]. Both expandable graphites were intercalated with sulfuric acid and had a bulk density of less than 1.2 g/cm^3^.

### 2.2. Test Procedure and Characterization

Preliminary testing of fire retardants in regards to miscibility and reaction kinetics (working life) with epoxy resin was initially performed. The working life is defined as the duration in which a mixture of epoxy resin and hardener is still workable, i.e., the viscosity and temperature still allow the application to a substrate or part. Both temperature and viscosity increase during the curing process. The influence of the addition of fire retardants on the working life of the epoxy was investigated by heat development during the exothermic curing process in ambient conditions. Specimens with 78 g of two-component epoxy resin and 7.8 g (10 wt.-% of epoxy) flame retardant were mixed for five minutes in an 80 mm diameter polypropylene cup. A thermo-element was then placed vertically in the center of each specimen to record the temperature over time. Based on the results, the mixture configurations were concluded by eliminating fire retardants that limited the working life by more than 10% compared to the reference resin. Moreover, fire retardants, of which less than 30 wt.-% in relation to the epoxy resin was dispersible, were excluded from further investigation. The dispersibility was measured by the evaluation of residing particles at the bottom before curing was completed. In the following step, a statistical test plan was designed concerning suspected interactions of components and restrictions.

### 2.3. Design of Experiments

For the test set-up, a randomized D-optimal design with a reduced quartic model was used to address different levels of interaction between the components [24]. The mixture design for the five components, numbered A to E as specified in Table 1, was based on several restrictions. The maximum weight ratio of 30 wt.-% in regards to the total mixture for fire retardants was chosen to emphasize the effects of different fire retardants whilst keeping a fire-retardant polymer system possible to be used for fiber-reinforced composites. Due to the costs of fire retardants and possible negative effects on mechanical properties, a maximum amount of 30 wt.-% of fire retardants in the mixture was chosen [9,25]. The mixture design allowed for all combinations of fire retardants in one mixture, provided that the total relative weight of fire retardants did not exceed 30 wt.-% of the total mixture.

(1) $$\underset{i}{\sum }X_{i}=100\%$$

(2) $$\begin{array}{c} \sum X_{EP}+X_{APP}+X_{EG}+X_{ATH}+X_{MEL}=100\%\\ X_{EP}\geq 70\%\;;\;\forall \;X_{i}\neq \;X_{EP}\;\leq 30\% \end{array}$$

The design of the experiment was set up so that synergistic or antagonistic effects could be observed. Synergistic effects mean a significant increase in fire retardancy response due to the combination of fire retardants compared to mixtures, including a single fire retardant at the same weight ratio [26]. Opposed to this, antagonistic effects mean a significant reduction of said response due to the combination of fire retardants.

**Figure 1:**
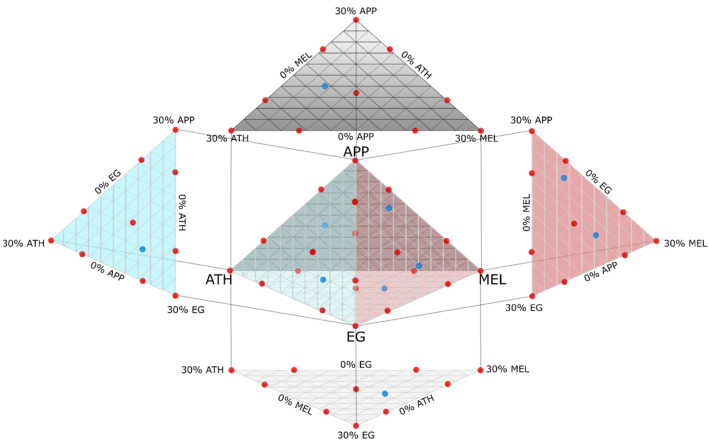
Four-dimensional test space, the relative amount of fire retardants orthogonally to one another. Corners show the maximum loading content of selected fire retardants. APP: ammonium polyphosphate; ATH: aluminum trihydroxide; MEL: melamine; EG: expandable graphite. The fifth dimension of epoxy resin is not shown. Red dots: designed model and replicate points; blue dots: randomly distributed lack-of-fit-point.

The design points in the test space defined the mixtures that were consecutively produced and tested in the LOI test. The OI levels for each mixture were integrated as a response in the DoE, and a Sheffé regression was calculated to estimate the values of the remaining test space [27]. A special quartic mixture order was chosen to include possible dependencies between ingredients. To eliminate statistically insignificant model terms (*p* > 0.05), a backward selecting algorithm based on Akaike’s Information Criterion for small sample sizes (AICc) was used [28]. Thus, only terms that have a significant share in explaining the model remain. This result was then adjusted by re-adding eliminated model terms necessary for the hierarchy of the model. For example, the content of ingredient “A” might not play a significant role in explaining the overall behavior but because of its synergistic effect with “B”, the model term “AB” is significant. For the hierarchy of the model, it is then necessary to include “A” again despite its statistically insignificant effects as a singular ingredient. Based on the regression model, the arithmetical optimum mixture was produced and tested. Figure 1 demonstrates the test space upon which the model was built.

### 2.4. Production of Fire-Retarded Epoxy Polymer and CFRP-Samples

**Figure 2:**
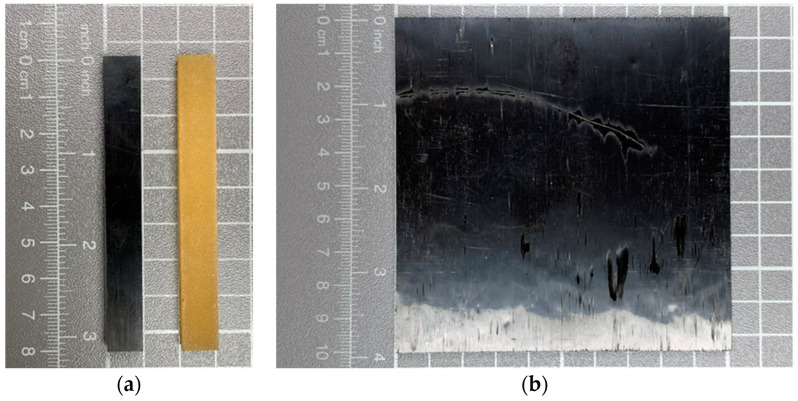
Samples used in the fire performance investigation: (**a**) Limiting oxygen index test sample, with carbon fibers on the left and without carbon fibers on the right (**b**) Cone calorimetry sample of a carbon fiber-reinforced polymer with 10 layers of unidirectional fabric.

For each specimen type, between 211 g and 302 g of epoxy resin and between 54.9 g and 78.4 g of hardener, depending on the ranges shown in Table 1 with a constant mixing ratio of 100:26, were used. Additionally, fire retardants were added based on the results of the DoE analysis presented in Section 3.2. To delay the curing reaction, fire retardants were first mixed with the hardener in a dissolver (Dispermat CV-SIP-Timer, VMA Getzmann GmbH, Reichshof, Germany) for 5 min at 1000 rpm. Due to the low viscosity of 0.15–0.2 Pa∙s of the hardener compared to the resin and mixed system, less agglomeration of particles was expected [29]. Afterward, the resin was added and the mixing process was continued for another 5 min. The final mixtures were cast in silicone molds with the dimensions of 80 mm × 10 mm × 4 mm for LOI test specimens following DIN EN ISO 4589-2, as shown in Figure 2a [30]. Deviating from ISO 5660-1, the cone calorimetry samples were cast on a 2 mm wooden carrier plate made of poplar to prevent excessive dripping temperatures above the glass transition temperature (Tg) of 59 °C during the test [29,31]. The carrier plate was placed at the bottom of a silicone form and resin was cast with the dimensions of 190 mm × 190 mm and a thickness of 4 mm. After curing, specimens were cut out of the center to the size of 100 mm × 100 mm, as shown in Figure 2b.

Specimens containing carbon fabrics were produced in a hand layup process layer by layer. After an initial layer of one-eleventh part of the resin, layers of carbon and resin were alternately placed and worked in with a plastic roller. The 4 mm thick, 10-layer CFRP samples contained a fiber volume fraction of 49.3%. After 24 h of curing at room temperature, all specimens were cut out according to the aforementioned sizes and conditioned at 20 °C/65 RH for a minimum of two weeks before testing.

### 2.5. Test Methodology

#### 2.5.1. Limiting Oxygen Index (LOI) Test

The limiting oxygen index test was carried out on a Dynisco Limiting Oxygen Index Analyzer (Franklin, TN, USA). After specimens with the size of 80 mm, 10 mm, and 4 mm were inserted, the concentration of oxygen within the device was adjusted to the desired test value by adding nitrogen, and the tube was flushed for 30 s. The specimens were exposed to a 3 cm methane flame for 30 s in a top surface ignition, and the time until extinction was recorded. If the sample burned continuously for more than 180 s or the flame burned more than 50 mm in length, the test was considered failed. Iteratively, the oxygen content was examined and repeated twice for the highest possible value passed.

#### 2.5.2. Thermogravimetric Analysis

The thermal stability of the epoxy mixtures was investigated with thermogravimetric analysis using the Thermal Analyzer TGA/DSC 1 STARe System (Mettler Toledo AG; Greifensee, Switzerland). Each specimen of approx. 10 mg was taken from the same batches of LOI samples and placed in an aluminum oxide crucible. The chamber containing the test specimen was heated from 25 °C to 1000 °C, at a heating rate of 10 °C/min under a constant airflow of 50 mL/min (40 mL/min N_2_, 10 mL/min O_2_).

#### 2.5.3. Cone Calorimetry

In addition to the LOI test, a cone calorimeter (Fire Testing Technology Limited, East Grinstead, UK) was used to gain additional information on the evaluation of the fire risk [32]. The cone calorimeter was adjusted to 50 kW·m^−2^ as recommended in ISO 5660-1 and calibrated beforehand to minimize the influence of the test equipment and environmental factors. A weight change of the specimen, oxygen, and carbon dioxide in the exhaust was recorded and used to calculate total, average, and peak heat release rates. The heat release rate (HRR), average heat release rate over 180 s (HRR_180s_), peak heat release rate (pHRR), and total heat release over the duration of ten minutes (THR_600s_) was chosen for evaluation to represent the fire performance in terms of the contribution to fire growth [33].

#### 2.5.4. Test Sequence

To reduce possible interferences, the limiting oxygen tests were carried out without carbon fibers at first. After evaluation of the effects of all fire-retardant mixtures, those with an OI value of at least 30 vol.-% were considered for further investigation, thus, conducted again with carbon fiber reinforcement and investigated in the cone calorimeter. The regression analysis was carried out to optimize the mixture composition. An auxiliary thermogravimetric analysis was performed on all substances used to obtain thermal stability and temperature-dependent degradation behavior.

## 3. Results and Discussion

### 3.1. Pre-Screening of Fire Retardants

**Figure 3:**
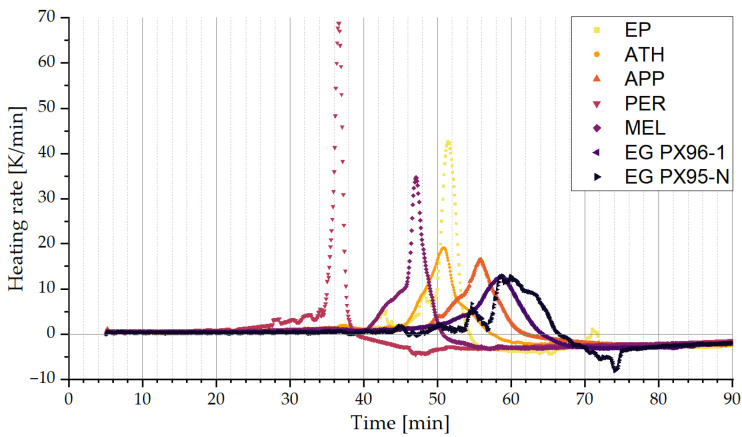
Temperature change ∆T over time due to exothermal curing reaction (EP: pure epoxy; other samples abbreviated by additional fire retardant. ATH: aluminum trihydroxide; APP: ammonium polyphosphate; PER: pentaerythritol; MEL: melamine; EG: expandable graphite).

As discussed in Section 2.2, a pre-screening of possible fire retardants was conducted. The temperature development of the epoxy systems, which were monitored by thermocouples, are shown in Figure 3. The addition of 10 wt.-% of Pentaerythritol (PER) shows a significant shift in the curing behavior, as it not only decreases working life but also increases temperature increments significantly. A less strong but still accelerated reaction performance can also be found for melamine (MEL). For other fire-retarded epoxy systems, the addition of fire retardants can be described as dilutive, due to their addition of heat capacity but otherwise with low or no influence on the reaction performance. PER and MEL, however, act as catalysts in the curing reaction between hardener and resin, thus accelerating heat generation and limiting working life by 29%. In consequence, because of its strong catalytic effect, PER was eliminated from further investigations. Although MEL triggered faster curing; as shown in Figure 3, the reduction of working life by 8% was considered neglectable.

The miscibility of fire retardants was tested by the dispersion of high loading ratios into the uncured epoxy resin matrix. If signs of segregation occurred, i.e., visible deposition at the bottom of the cured resin, fire retardants were excluded from further investigations. As shown in Table 2, most of the ingredients could easily be incorporated into the mixture to higher contents than the set limit of 30 wt.-%. Only one fire retardant, the commercially available expandable graphite (EG) PX95-N with coarser composition (min. 70% >300 µm), was showing clear signs of deposition in the mixture before curing and therefore excluded from further testing. The other type of EG, PX 96/-1, however, dispersed more easily due to its finer particle size distribution (80% <150 µm). No signs of visible deposition during or after curing in epoxy resins was found for APP, ATH, MEL, and PER at loading levels of 30 wt.-%.

### 3.2. Design of Experiments

Following the limits of the test set-up described in Section 2.3, the factorial test plan was set up as presented in Table 3. The set-up included three types of points, i.e., model, replicate, and lack-of-fit points. Model points were chosen to represent the experimental test space with a minimum number of trials. Replicate points were used to evaluate the accuracy of the test and the production process factors, i.e., how large the variation within the design was by comparing the performance of identical mixtures which were produced and tested at separate times. Lack-of-fit points produce additional information on the discrepancy between the regression model and observed values. The order of the specimen production and testing was statistically randomized as stated in the column “Run order” to minimize overshadowing effects. The last three test points (40 to 42) were added after the test set had been finished to verify the resulting regression model.

### 3.3. Limiting Oxygen Index

**Figure 4:**
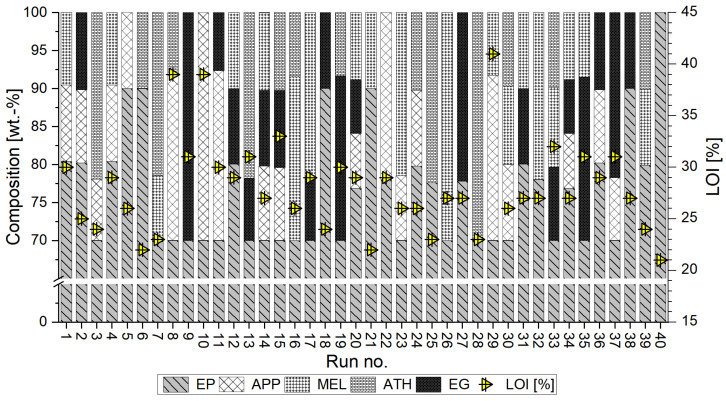
Weight percentage of fire retardants (left scale) and resulting limiting oxygen index levels (right scale) of epoxy resin samples in run order. Patterns of columns represent the weight percentage of mixture components. Yellow triangles represent LOI. Run no. 40 added as a reference sample of pure epoxy resin.

The results of the limiting oxygen index test as well as the composition of each sample are juxtaposed in Figure 4. The limiting oxygen index (LOI) of the reference sample (run no. 40) amounted to 21% and represents the lowest oxygen fraction of the groups, above which the sample would continuously burn for more than 180 s or burn on more than 50 mm of the total height. The partial substitution of the epoxy resin system by fire retardants (FR) expectedly led to an increase in the oxygen index level. Only one mixture (run no. 1) with 20% or less FR reached an LOI level of 30%. It contained equal parts of APP and MEL. Specimens containing APP as main FR (run no. 8, 10, 11, 22, 29) also achieved the highest LOI levels among the tested mixtures. The composition of 70 wt.-% EP and 30 wt.-% APP (run no. 10) resulted in an OI of 39%, which was only exceeded when one third of APP was substituted by MEL. Run no. 29 resulted in the maximum LOI level of 41% among the tested samples.

**Figure 5:**
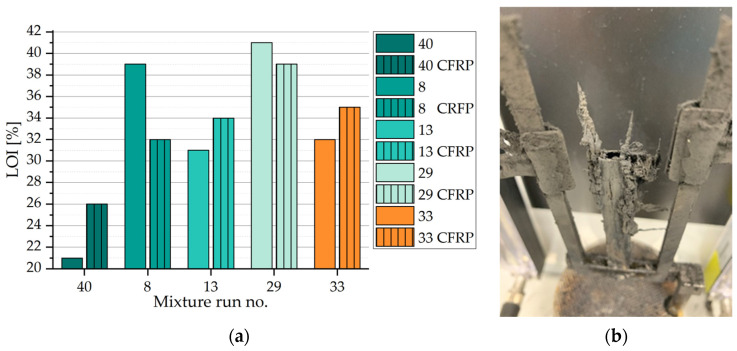
Limiting oxygen index test results and sample: (**a**) Juxtaposition of LOI levels of polymer and CFRP samples sorted by run no. (40: Reference, w/o FR; 8: EP with APP and ATH; 13: EP with EG and ATH; 29: EP with APP and MEL; 33: APP, MEL, and ATH); striping indicates carbon fiber reinforcment; (**b**) CFRP sample after flame exposure with soot pinnacles due to wicking and visible gap between layers of carbon fabric.

In the course of further analysis, a regression model was built to predict untested values within the design space. Additionally, for the reference and selected well performing mixtures, CFRP samples with the same weight ratios of FR to the matrix were tested and juxtaposed with the respective polymer samples. Since the modes of actions of the fire retardants are different, e.g., APP and MEL work inter alia as spumific agents, the fire retardation could be hindered by carbon fibers. Therefore, the selection was based not only on the performance of polymer samples. Mixtures with the run no. 8 and 29, both containing high amounts of APP, were selected for their performance. Whereas, mixtures with the run no. 13 and 33 were included as an extension of the variety of fire retardants. Figure 5a shows the results of the selected CFRP samples in comparison with polymer samples with the same FR to EP ratios. For the reference mixture without any FR, CFRP showed a significantly increased LOI level of 26% compared to 21%. A similar trend was visible in mixture run no. 13 and no. 33, where the CFRP sample also showed an increased LOI level by 5 and 3 percentage points compared to the respective polymer sample. A contrary effect was recorded for sample groups with the run no. 8 and no. 29. Here, the carbon-fiber-reinforced polymer samples showed a reduction in the LOI level of 7 and 2% points compared to their respective polymer samples. Carbon in the form of graphite is thermally stable compared to the aliphatic epoxy polymer. An increased limiting oxygen index, as found in the sample groups no. 40, 13, and 33 would, therefore, be expected for all CFRP specimens. However, two secondary effects have been found to influence the inflammability of CFRP samples. Firstly, a wicking of carbon fiber bundles was observable. It accelerated the flame spread along the height of CFRP samples. Figure 5b exemplarily shows a reference sample (run no. 40) after flame exposure at a high oxygen level. It failed the test inter alia due to strong wicking effects, where the polymer matrix between layers of carbon fiber fabrics was transferred to the top. After the test, soot pinnacles rise from the top of the specimen and a gap between the fabrics becomes apparent. Secondarily, for samples containing fire retardants where the course of action highly depends on volume increase, such as for APP, EG and MEL samples, carbon fibers restrict the course of action by mechanically hindering the expansion of the matrix system. Although expandable graphite has a similar cause of action, it does not form a closed surface; instead, singular platelets increase in volume, which reduces the latter effect for samples containing expandable graphite.

**Figure 6:**
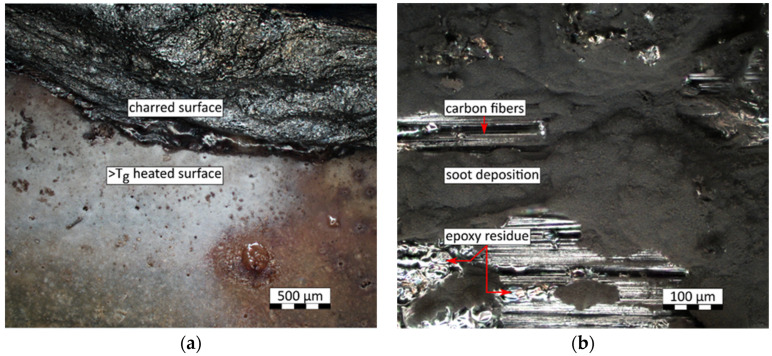
Optical microscopy of sample surfaces after limiting oxygen index test: (**a**) Transition area between reacted and unreacted APP and MEL embedded in the epoxy matrix (Run no. 29). (**b**) Epoxy resin sample w/o FR (Run no. 40). Few residues of polymer matrix around exposed carbon fibers and soot deposits.

In Figure 6a, the transition area of a flame-treated specimen under an optical microscope is shown. At the bottom, the APP- and MEL-containing specimen’s surface is smooth and only an accumulation of vesicular surface structures show that the polymer matrix exceeded the glass transition temperature. Closer to the heat source, distinct areas of foamed and charred residue are visible in the top region of the extended depth of the field-picture. Figure 6b, in contrast, shows a flame-treated specimen without fire retardants, in which epoxy mostly burned, exposing the carbon fibers underneath areas of soot deposition.

LOI results from the polymer testing were statistically analyzed to build a regression model that enables the prediction of LOI values for the design space described in Section 2. After the removal of statistically insignificant terms, a reduced cubic model, including terms to the third order, resulted from the analysis of the results [34]. It allows for the analysis of the hypothesis examining whether the fire retardants influence the fire performance measured by the limiting oxygen index of epoxy resin, and to what extent.

The resulting model terms strongly indicate that the correlation between APP and MEL as well as between EG and ATH take a significant role in the explanation of the fire performance in the LOI test, which is shown by the analysis of variances in Table 4. The overall model results in a *p*-value < 0.0001, covering eight degrees of freedom. Four of these result from the linear mixture, and are, hence, single mixture components. The combination of ingredients representing synergistic or antagonistic effects is reflected in second- and third-order terms, e.g., APP∙EG. Although the combination of APP and MEL shows a *p*-value higher than 0.05, it was re-added for the model hierarchy, since the third-order term APP∙MEL∙(APP-MEL) proved to be significant for the model. The latter describes the synergistic effect between both ingredients and incorporates the difference of both amounts in relation to each other. The fit statistics including R^2^, adjusted R^2^, and predicted R^2^ were calculated on this basis and are shown in Table 5.

The fit statistics reflect the model regression accuracy of the selected model shown in Equation (3). The correlation (R^2^) to the data points amounts to 0.8233. The adjusted R^2^ penalizes an increasing number of model terms or predictors and results in a lower value of 0.7777 but, therefore, reflects the quality of the correlation model more accurately. The predicted R^2^ is calculated by the measures of how well the model predicts a response value based on the lack-of-fit points incorporated in the model design. The difference of 0.127 between the predicted R^2^ and adjusted R^2^ can be considered to be in reasonably good agreement, i.e., the difference is less than 0.2 [34]. Adequate precision is a parameter for the signal-to-noise ratio. Values of greater than 4 indicate an adequate signal.

The model shown in Equation (3) was developed to calculate the limiting oxygen index level of a mixture based on its composition in weight percentage of each component. Its fit parameters are calculated on the basis of the boundary conditions, and extrapolations into higher contents of FR were not intended. The model consists of nine parameters, for which nine constants were calculated by a regression analysis. Five (a_1_ to a_5_) of the nine constants reflect the level of a singular effect of a constituent. Of the four fire retardants, the singular effect size of ATH (a_5_) is the lowest. The substitution of epoxy to the maximum amount resulted in a minor increase in the LOI level from 21% to 23%. If only a single fire retardant is to be used to improve the LOI, the FR with the highest (positive) effect size should be chosen. The effect size of single fire retardants increases from a_5_ (ATH), a_4_ (MEL), a_3_ (EG) to a_2_ (APP). In combination of two fire retardants, additional synergistic and antagonistic effects need to be considered and are accounted for by the four parameters (a_6_ to a_9_). a_6_ shows an antagonistic effect between APP and EG. Both FR are spumific, i.e., they increase the volume. Expandable graphite has shown to strongly increase in volume but not form a closed surface layer. APP, in contrast, releases gas and induces charring through the release of phosphoric acid, which forms a closed surface layer. This hinders EG to expand freely to the same extent as without APP. The constant a_7_, although statistically not significant, was re-added for model hierarchy. Considered isolated, the constant shows a small antagonistic effect of APP and MEL. However, it needs to be evaluated in combination with a_9_. Both factors combined reflect the overall strong synergistic effect of APP and MEL. a_8_ reflects a synergistic effect of ATH and EG.

(3) $$\begin{array}{c} \mathrm{LOI}\;\left(\%\right)={\left(a_{1}X_{EP}+a_{2}X_{APP}+a_{3}X_{EG}+a_{4}X_{MEL}+a_{5}X_{ATH}+a_{6}X_{APP}X_{EG}+a_{7}X_{APP}X_{MEL}+a_{8}X_{EG}X_{ATH}+a_{9}X_{APP}X_{MEL}\left(X_{APP}-X_{MEL}\right)\right)}^{2}\\ X_{EP}\geq 70\;wt.-\%\;;\;\forall \;X_{i}\neq \;X_{EP}\;\leq 30\;wt.-\%\\ a_{1}=\;4.623;\;\;\;\;\;\;\;\;a_{2}=9.772;\;\;\;\;\;\;\;a_{3}\;=7.847;\;\;\;\;\;\;a_{4}=\;6.878;\;\;\;\;\;a_{5}=5.043\\ a_{6}=-21.752;\;\;\;\;a_{7}=-3.751;\;\;\;\;a_{8}=25.848;\;\;\;\;\;a_{9}=193.9 \end{array}$$

**Figure 7:**
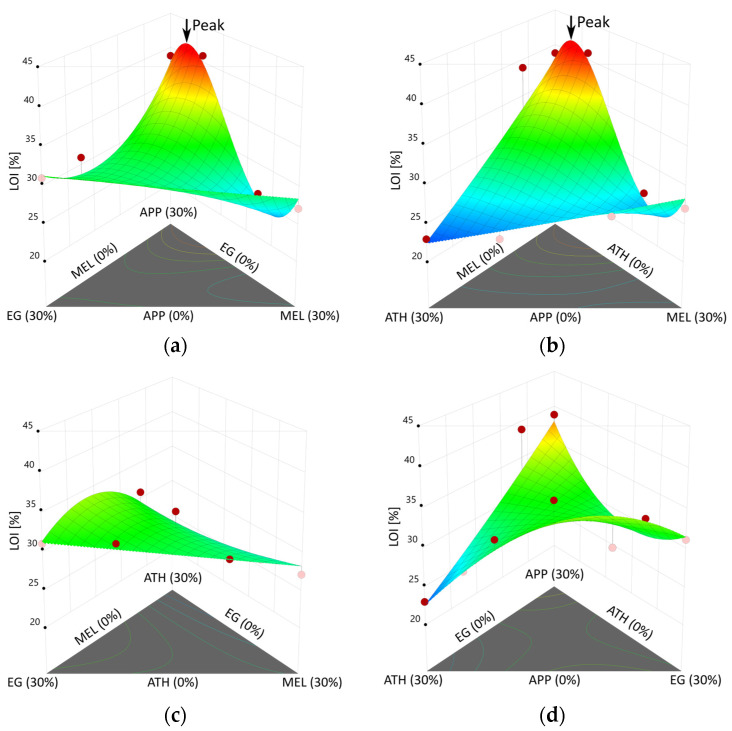
Response surface diagrams of limiting oxygen index results (dots: run points tested above (red) and below (pink) the response surface; surface: regression model). (**a**) Response surface for mixtures containing APP, MEL, and EG. (**b**) Response surface for mixtures containing APP, MEL, and ATH. (**c**) Response surface for mixtures containing ATH, EG, and MEL. (**d**) Response surface for mixtures containing APP, ATH, and EG.

Due to the multi-dimensional test space, the model results are plotted in several 3D-surface diagrams shown in Figure 7. The z-axis shows the resulting limiting oxygen index level for a mixture containing a minimum epoxy resin content of 70 wt.-% and a maximum of three fire retardants at a time in a range from 0 to 30 wt.-%. All other fire retardants are set to 0%; therefore, only a few of the 40 model points are shown in the diagrams. Each corner shows the result of a mixture containing 30% of a single fire retardant. The opposite edge of a corner shows the opposite extrema of 0% of that specific fire retardant.

The resulting model proposes a local maximum within the design space for the combination of APP and MEL. Two model points close to the model maximum (run no. 10 and 29) have shown the highest LOI levels of the tested mixtures of 39% and 41%, respectively. The mixture of 70 wt.-% epoxy, 24.6 wt.-% APP, and 5.4 wt.-% MEL represents the arithmetic maximum of the model, marked with the arrow “Peak” in the top two diagrams of Figure 7. Calculating the LOI response by Equation (3) yields a value of 42%.

Samples with that mixing ratio were produced and resulted in an LOI level of 45, the highest level of all tested specimens, exceeding the model calculation of 42.

The maximum in the LOI response indicates the importance of balancing spumific and charring agents in intumescent systems. During thermal decomposition, melamine (MEL) releases 10.4 times as much ammonia than the ammonium polyphosphate (APP) used in this study and hence, strongly contributes to the volume increase and dilution of the gaseous phase at the pyrolysis zone. APP, additionally to the release of ammonia, dehydrates carbon-containing compounds due to its decomposition to phosphoric acid (H_3_PO_4_). Thus, it induces a charring of the surface. In combination, the insulation layer becomes more voluminous than for mixtures containing only APP and more resistant to further heat-induced decomposition than for mixtures containing only MEL.

The results were used as a basis for the selection of material mixtures for further investigations in the cone calorimetry and fire resistance test.

### 3.4. Thermogravimetric Analyis

**Figure 8:**
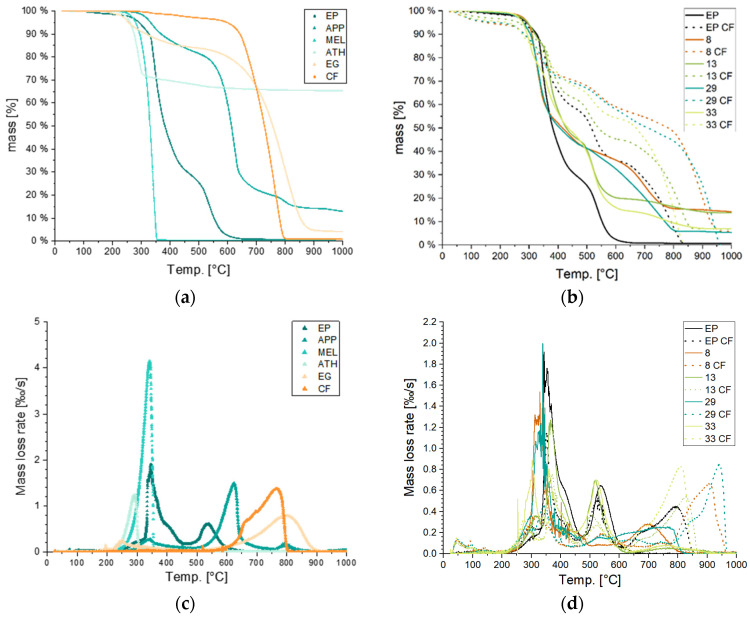
Thermogravimetric analysis of components and selected mixtures. (**a**) Mass over temperature of single components. (**b**) Mass over temperature of selected mixtures named after run number; CF: carbon-fiber-reinforced. (**c**) Mass loss rate of components. (**d**) Mass loss rate of selected mixtures.

The thermogravimetric analysis was conducted to define the decomposition temperatures of the materials. As shown in Figure 8 and Table 6, the reference sample of pure epoxy resin decomposed in two stages; a first stage with a peak at a temperature of 348 °C and a total weight loss of 70.9% and a second stage with a peak at 535 °C with a further weight loss of 28.8%, respectively. The decomposition finished at a temperature of 650 °C with only minor weight loss at further heating, resulting in a final residue of 0.13%. APP also showed two stages in the loss rate with a first peak at around 335 °C with a combined loss of 17.7%, which was related to the elimination of ammonia and water vapor. The remaining ultraphosphate decomposes in a second stage with a peak at 624 °C with a 58.9% weight loss. The final residue of phosphoramidic and phosphorimidic groups accounts for 17.42% [35]. ATH shows a single weight loss rate maximum at 290 °C, resulting in a weight loss of 29.2%. Under the elimination of water vapor, thermally stable aluminum oxide is formed. After a continuous reduction in another 5.29%, a residue of 65.5% remained. EG decomposes in two steps. A first peak at around 245 °C indicates the decomposition of the acidic intercalation, forming gases that are responsible for the exfoliation of the EG. The second peak recorded at around 800 °C is attributed to the oxidation of graphite and accounts for a weight loss of another 80%. MEL decomposes rapidly in a single step with a peak mass loss rate at 340 °C mainly into ammonia leaving no residue. CF retains mass for the longest heating time. Only small weight losses can be observed, which are believed to be due to sizing removal and decomposition of polyester stitching. Other than this, CF are thermally stable until 600 °C with a peak in the mass loss rate (MLR) at 761 °C, leaving a neglectable residue of less than 1%.

Inevitably, the thermal decomposition behavior becomes more complex for mixtures of epoxy resin and CFRP in combination with multiple fire retardants. As shown in Table 7, the distinguishable peaks in MLR of the single components are identifiable, and mass losses are respective to the weight ratio differences between epoxy resin and CFRP samples. For example, the CFRP sample composed of relatively equal weight amounts of epoxy resin and carbon fibers shows identical peaks with epoxy resin at around 365 °C and 535 °C, as well as the peak mass loss of carbon fibers at 759 °C. Contrary to this, in sample no. 8 and 8 CF, the first decomposition peak of ATH smoothly transitions into the mass loss peak of APP, and they are no longer distinguishable. Interestingly, although expandable graphite (EG) has the highest decomposition temperature, the two highest peak temperatures are found for specimens not containing EG but other fire retardants with lower decomposition temperatures. The addition of APP and ATH for no. 8 CF as well as APP and MEL for no. 29 CF results in peaks around 860 and 885 °C, respectively. These decomposition temperatures are assigned to carbon fibers in the mixtures.

Figure 8 shows the thermal decomposition processes in the TGA under a normal atmosphere containing 21% of oxygen at a heating rate of 10 K/min. On the top left, the mass over temperature of single components is presented. Underneath, the respective mass loss rate (MLR) of the samples is presented. On the right, selected mixtures were analyzed under identical conditions. The epoxy resin sample was chosen as a reference to compare with the two highest scoring mixtures on the LOI analysis (run no. 8 and run no. 29). The top right diagram shows the thermal degradation behavior of those mixtures by a continuous line for mixtures without and by a dashed line for mixtures including carbon fibers. In the bottom right, the respective mass loss rates of these samples are shown.

As decomposition is related to the chemical altering of the material’s molecules, mass changes occur. In the case of thermal degradation, as explained before, pyrolysis is accompanied by a release of volatiles into the gas phase and therefore, causes mass losses. As visible in Figure 8a,c, all fire retardants except for ammonium polyphosphate show more pronounced mass losses before the first thermal decomposition stage of the epoxy resin system. The incorporation of CF into the epoxy matrix system resulted in prolonged mass retention at equal temperature levels for all samples. By substituting 30 wt.-% of the epoxy resin matrix by fire retardants, e.g., in specimen no. 29 with 21.8% APP and 8.2% MEL, and no. 8 with 21.8% APP and 8.2% ATH, the mass loss rate (MLR) in the temperature range of 400 to 800 °C decreased significantly. This reflects the rather high temperatures necessary to further decompose ammonium polyphosphate. While the reaction enthalpy of aluminum trihydroxide was not reflected in the thermogravimetric analysis due to the nature of the test, higher mass retention can be observed to be caused by the formation of aluminum oxide.

From the different decomposition temperatures, it can be concluded that fire retardants have to be carefully selected if structures are very thin. If heat is quickly distributed through the structure (thin films for example), the epoxy resin system may already decompose thermally before temperatures are high enough to decompose the fire retardants such as APP.

### 3.5. Cone Calorimetry

**Figure 9:**
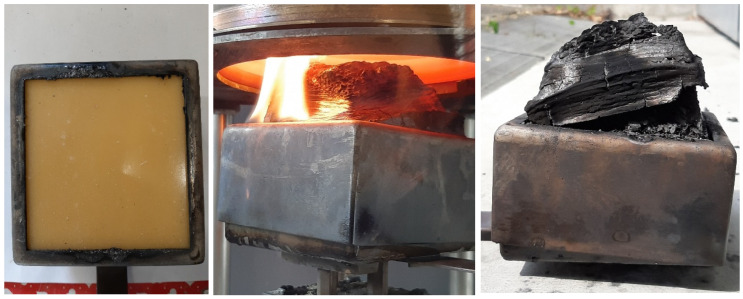
Cone calorimetry sample before (**left**), during (**center**), and after test (**right**) of an intumescent fire-retarded specimen.

Samples with an OI of 30 and above and a reference sample of pure epoxy were selected for additional investigation in the cone calorimeter. Figure 9 demonstrates a representative sample of mixture no. 8, consisting of epoxy polymer with 22 wt.-% APP and 8 wt.-% ATH. During exposure in the cone calorimeter, the intumescent behavior becomes clearly visible. The decomposing APP leads to a charring of the surface, while the release of gas causes volume expansion, which decreases the density. Both effects combined decrease the heat transfer to the condensed phase as well as mitigate volatiles into the combustion zone, thus reducing the heat release rate.

**Figure 10:**
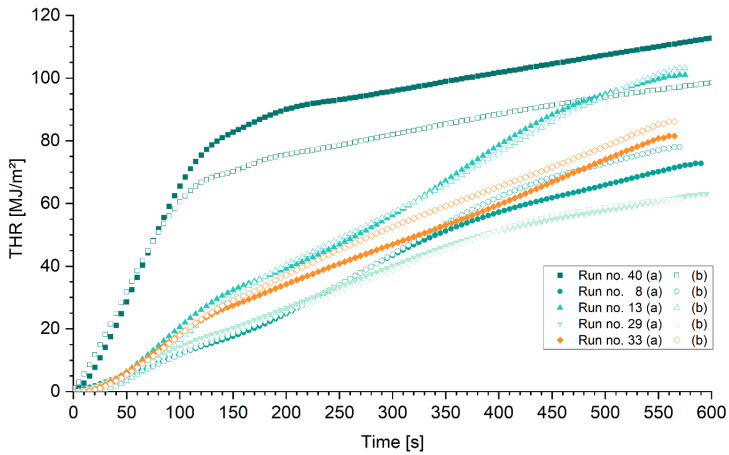
Total heat release over time of selected polymer mixtures. Run no. 40—reference sample of epoxy resin without fire retardants; no. 8: Ammonium polyphosphate (APP) and aluminum trihydroxide (ATH); no. 13: ATH and expandable graphite (EG); no. 29: APP and Melamine (MEL); no. 33: MEL, ATH, and EG; Mixtures (run no.) grouped by color and symbol.

**Figure 11:**
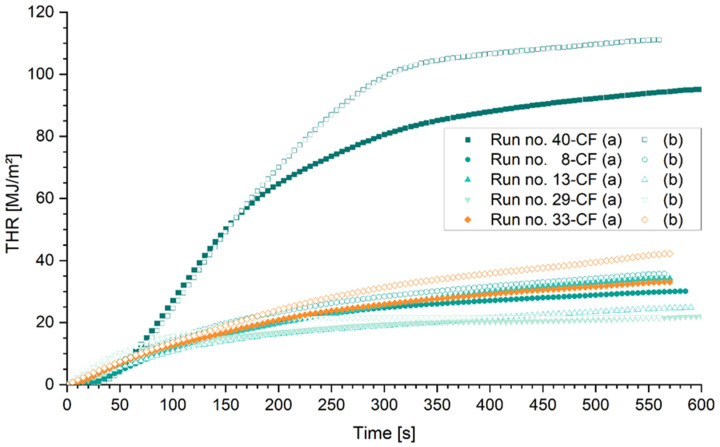
Total heat release (THR) over time of selected CFRP-mixtures: Run no. 40—reference sample of epoxy resin without fire retardants; no. 8: Ammonium polyphosphate (APP) and aluminum trihydroxide (ATH); no. 13: ATH and expandable graphite (EG); no. 29: APP and Melamine (MEL); no. 33: MEL, ATH, and EG; mixtures (run no.) grouped by color and symbol.

The total heat release reflects the calorific value of a material or product as well as its mass. The diagrams presented in Figure 10 and Figure 11 show the total heat release (THR) over time for the duration of 600 s of samples heated with a 50 kW/m^2^ cone radiator in a horizontal setup. Figure 10 shows selected polymer samples without (run no. 40) and with various combinations of fire retardants (run no. 8, 13, 29, and 33), respectively. Without fire retardants, the heat release of epoxy is higher than of the comparative samples. After 100 s, 60 and 65 MJ/m^2^ have been released by the two specimens of run no. 40. The heat release can be divided into two distinct domains with a transition between them. There was a fast ignition and high heat release rate from the point of ignition until approximately 120 to 150 s and another almost constant heat release rate afterwards until the test was stopped. This first stage shows the high inflammability and contribution of the untreated epoxy resin to rapid fire growth. For polymer samples containing fire retardants, the heat release develops slower with changing heat release rates over time. Expectedly, the total heat released after 600 s is lower if samples contained fire retardants, compared with the epoxy sample (run no. 40 b). The difference between run no. 40 (a) and (b) could be explained by the variation in the total mass of 14.9%. The specific total heat release for both specimens amounts to 2.5 MJ m^−2^ g^−1^. The specific total heat release for all selected samples is given in Table 8.

Figure 11 presents the total heat release (THR) of CFRP samples with identical weight ratios of the fire retardant to epoxy resin system. Only small differences are found in the total heat release between the untreated CFRP and the untreated polymer samples. However, if the sample weight is considered, the difference becomes apparent.

**Figure 12:**
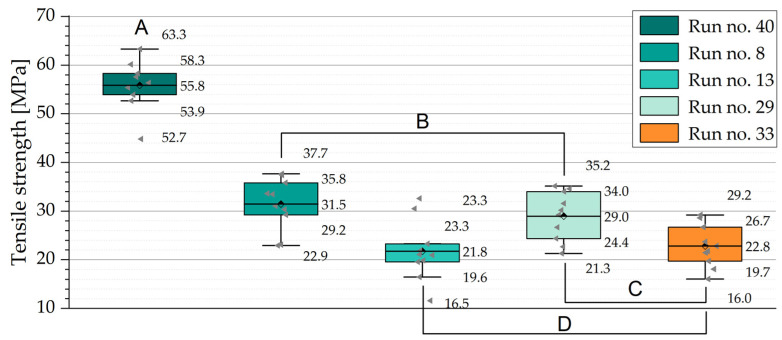
Boxplot diagram of the tensile strength of selected polymer mixtures with fire retardants. Values describe maximum, 75th percentile, mean, 25th percentile, and minimum. Results outside of 1.5 IQR are treated as outliers. Letters A to D indicate grouping by Tukey test. Statistically significant difference in means is found in groups not sharing a letter.

The specific total heat release over 600 s of neat epoxy polymer results in 2.47 MJ·m^−2^·g^−1^ for the respective CFRP in only 1.78 MJ·m^−2^·g^−1^; hence, it has an average reduction of 28%. The reduction is related to the substitution of flammable polymeric matrix by comparatively thermally stable carbon fibers. It is suspected that the substitution of the same amount of epoxy by carbon fibers in CFRP specimens causes equal changes to the specific THR. Due to the limited number of specimens, the samples are divided into two groups for the statistical analysis. The reference group contains four specimens; the fire-retarded specimens are treated together as a second group. At an alpha level of 0.05, the hypothesis that the population means of both sample groups are equal cannot be accepted. The mean differences between mixtures containing fire retardants and the respective carbon-fiber-reinforced polymer mixtures are significantly higher than the difference between the epoxy sample and CFRP sample without fire retardants. As shown in Figure 12, the mean difference for fire-retarded CFRP samples compared to their polymer equivalent amounts to 51.1%, whereas the mean difference of non-fire retarded samples amounts to 27.9%. Interestingly, the reduction in spec. THR_600s_ is larger in fire-retarded samples then the carbon-fiber weight percentage. The average heat release over 180 s (HRR_180s_) shows different results. For the mixture run no. 8, which contains APP and ATH as well as for mixture run no. 33, which contains MEL, ATH, and EG, the average heat release reduces to a similar extent as the spec. THR_600s_. The already low average heat release of around 100 kW/m^2^ of mixture run no. 8 and 29 does not reduce to the same extent. The carbon-fiber-containing reference sample (run no. 40) without fire retardants releases on average around 16% less heat than the respective polymer sample. The reason for the main differences between the total heat release and heat release rate comparison of carbon fiber containing samples and polymers is found in the heat release development after the first peak. The heat release rate of carbon-fiber-reinforced samples drops significantly after an initial peak and continuous to stay low, whereas polymer samples in general show a second peak of heat release. The time to ignition (TTI) is an indicator of the inflammability of a material. Neat epoxy resin took an average of 29 s to ignite. Any substitution of epoxy resin by fire retardants increased that duration. Significant differences were found, ranging from an average increase of 10.3% for run no. 8 up to 37.9% for run no. 13. A different picture emerges for the carbon-fiber-containing specimens. The time to ignition of pure CFRP samples increased to an avg. of 38 s (+28.1%) compared to the neat polymer; whereas, for fire-retarded specimens, a decrease between 15.6 and 35.4% was observed. A possible explanation for the contradictory effect is assumed by the authors to be surface roughness. Although not tested within the scope of this investigation, the increased viscosity of fire-retarded resin did show less workability, especially in combination with fabrics. The peak productions of carbon oxides are represented by pCOP for carbon monoxide and pCO_2_P for carbon dioxide. Both coincide and correlate moderately with the peak heat release (pHRR), with a coefficient of determination of 0.63 and 0.78, respectively. The highest rates of carbon oxide production as well as heat release are found for neat epoxy resin. The substitution with fire retardants reduced the pHRR by an average (CoV) of 68.8% (11.4%), pCOP by 68.6% (8%), and pCO_2_P by 75.1% (12.3%) for polymer samples. In CFRP samples, the reduction similarly amounts to 70.7% (8.4%) on average for pHRR, 43.6% (46%) in pCOP, and 69.9% (7.4%) in pCO_2_P. This reflects the strong reduction in oxidative processes due to the substitution of the flammable aliphatic polymer by fire retardants. However, it needs to be pointed out that the power of these statistics is limited due to the small number of specimens.

Carbon-fiber-reinforced polymers are commonly used in load-bearing functions; therefore, the mechanical material properties are of consideration in the selection process of fire retardants for a polymer with enhanced fire performance. Figure 12 demonstrates the tensile strength of polymer samples without (Run no. 40) and with fire retardants. The tensile strength is significantly reduced for all specimens containing fire retardants. As indicated by the grouping letters, statistically significant differences can be found between all samples except the samples sharing a grouping letter at an alpha level of 0.05 using a Tukey test. On average, the loss in tensile strength amounted to 53% for all fire-retarded mixtures in comparison to neat epoxy. Among those, samples contained APP, as the main fire retardant (run no. 8 and run no. 29) showed the smallest reduction in the mean tensile strength of 48 and 44%, respectively. All reductions were significantly higher than the amount of epoxy replaced by fire retardants. A potential explanation is the distribution of fire retardants within the mixture. Accumulations of fire retardants locally reduce the fracture energy necessary and facilitate the local crack propagation, which results in lower strength.

## 4. Conclusions

An array of non-halogenic fire retardants was tested for the use in epoxy matrix systems and carbon-fiber-reinforced polymer (CFRP). After preliminary tests on miscibility and effects on the curing process of epoxy resin, a design of experiments was set up to optimize fire retardance based on limiting oxygen index (LOI) tests and cone calorimetry. The combined fire retardation effects of aluminum trihydroxide (ATH), ammonium polyphosphate (APP), melamine (MEL), and expandable graphite (EG) were compared and a regression model developed to describe the test space. From this, a new mixture ratio for the arithmetic maximum was derived and tested, verifying the calculated model response by achieving a limiting oxygen index of 45 for a mixture composed of a combination of 24.6 wt.-% ammonium polyphosphate and 5.4 wt.-% melamine. Samples containing exclusively one fire retardant resulted in lower LOI values and a higher average heat release rate (HRR_180s_); e.g., epoxy samples with 30 wt.-% of APP achieved an LOI of 39, and those with 30% of MEL achieved an LOI of 27. The strong synergistic effect between phosphorous and nitrogen and its dependency on the mixing ratio was underlined with the results. Although ammonium polyphosphate is composed of phosphorous as a catalyst and charring agent as well as ammonia as a spumific agent, the partial substitution of 18% of APP by melamine led to a further increase in the OI from 41 to 45, which is related to the 10.4 times higher release of ammonia by MEL in comparison to APP.

APP, mainly as a charring agent in combination with the spumific ammonia release of MEL, resulted in the best fire retardancy in terms of LOI (45), average HRR_180s_ (104 kW/m^2^), and spec. THR_600s_ (1.14 MJ·m^−2^·g) within the tested fire retardants for polymers. Moreover, for carbon-fiber-reinforced polymers, the combination of APP and MEL resulted in the highest performance of LOI (39) and spec. THR_600s_ (0.54 MJ·m^−2^·g). The average HRR_180s_ of 86.5 kW/m^2^ was only undercut by mixture run no. 13, containing aluminum trihydroxide and expandable graphite as fire retardants with an average of 74.5 kW/m^2^.

The transfer of fire retardancy results from a polymer to a fiber-reinforced polymer proved to be non-trivial. Partially negative effects for CFRP samples were observed both in the limiting oxygen index test as well as in the cone calorimetry. The mechanical restriction of fibers against volume increase is hypothesized as a potential reason for fire spumific retardants. In cone calorimetry, the diminishing distance between the sample surface and radiator of intumescent fire-retardant mixtures, i.e., APP- and MEL, has to be considered. The open porous surface of expandable graphite (EG) platelets was less likely to fall off from the exposed surface in the horizontal cone test compared to the vertical LOI test. In terms of the optimization of fire performance, attention has to be given to the drawbacks and limitations of each fire performance test in regards to the retardation principle as well as to the mechanical strength losses that are associated with the replacement of neat epoxy with fire retardants.

## Figures and Tables

**Table 1:** Mixture design for components, abbreviations, and their mixing ratios by weight.

Component	Name	Abbreviation/Subscript in Equation (2)	Product	Range (wt.-%)
A	Epoxy resin incl. Hardener	EP/$X_{EP}$	Ampreg 31+3X	70–90
B	Ammonium polyphosphate	APP/$X_{APP}$	AP 422	0–30
C	Expandable graphite	EG/$X_{EG}$	PX 96/-1	0–30
D	Melamine	MEL/$X_{MEL}$	Melafine	0–30
E	Aluminum trihydroxide	ATH/$X_{ATH}$	Micral 855	0–30
				∑ 100

**Table 2:** Miscibility test results and loading content tested without signs of visible segregation.

Type of Fire Retardant	Weight of Epoxy Resin [g]	Weight Ratio of Additive [g/g Epoxy]
APP	30.20	>30%
PER	30.35	>30%
MEL	30.40	>30%
EG PX 95-N	31.20	>30%
EG PX 96/-1	30.82	<10%
ATH	30.26	>30%

**Table 3:** DoE test set up with model, replicate, and lack-of-fit points. Std. order is the order of points produced by the model. These have been randomized to create the run order to reduce bulking effects and distribute replicate points.

Std. Order	Run Order	Design Point (Type)	EP (wt.-%)	APP (wt.-%)	EG (wt.-%)	MEL (wt.-%)	ATH (wt.-%)
14	1	Replicate	80%	10%	0%	10%	0%
1	2	Model	80%	10%	10%	0%	0%
37	3	Model	70%	8%	0%	0%	22%
13	4	Model	80%	10%	0%	10%	0%
21	5	Model	90%	10%	0%	0%	0%
31	6	Model	90%	0%	0%	0%	10%
35	7	Model	70%	0%	0%	9%	21%
26	8	Model	70%	22%	0%	0%	8%
10	9	Model	70%	0%	30%	0%	0%
7	10	Model	70%	30%	0%	0%	0%
8	11	Model	70%	22%	8%	0%	0%
15	12	Model	80%	0%	10%	10%	0%
36	13	Model	70%	0%	8%	0%	22%
25	14	Model	70%	10%	10%	10%	0%
33	15	Lack of Fit	70%	10%	10%	0%	10%
28	16	Model	70%	0%	0%	22%	8%
18	17	Model	70%	0%	8%	22%	0%
23	18	Replicate	90%	0%	10%	0%	0%
27	19	Model	70%	0%	22%	0%	8%
3	20	Model	77%	7%	7%	9%	0%
5	21	Model	90%	0%	0%	10%	0%
6	22	Model	78%	22%	0%	0%	0%
17	23	Model	70%	9%	0%	21%	0%
34	24	Lack of Fit	80%	10%	0%	0%	10%
38	25	Model	78%	0%	0%	0%	22%
20	26	Model	70%	0%	0%	30%	0%
24	27	Model	78%	0%	22%	0%	0%
39	28	Model	70%	0%	0%	0%	30%
11	29	Model	70%	22%	0%	8%	0%
29	30	Lack of Fit	70%	10%	0%	10%	10%
16	31	Replicate	80%	0%	10%	10%	0%
19	32	Model	78%	0%	0%	22%	0%
30	33	Lack of Fit	70%	0%	10%	10%	10%
4	34	Replicate	77%	7%	7%	9%	0%
12	35	Model	70%	0%	21%	9%	0%
2	36	Replicate	80%	10%	10%	0%	0%
9	37	Model	70%	8%	22%	0%	0%
22	38	Model	90%	0%	10%	0%	0%
32	39	Lack of Fit	80%	0%	0%	10%	10%
40	40	Additional	100%	0%	0%	0%	0%
41	41	Verification	75%	21%	0%	4%	0%
42	42	Verification	70%	25%	0%	5%	0%

**Table 4:** Analysis of variances of the cubic model with selected terms.

Source	Sum of Squares	df	Mean Square	F-Value	*p*-Value	Statistical Significance
Model	5.44	8	0.68	18.05	<0.0001	significant
Linear mixture	4.21	4	1.05	27.95	<0.0001	significant
APP·EG	0.29	1	0.29	7.72	0.0092	significant
APP·MEL	0.01	1	0.01	0.25	0.6215	not significant
EG·ATH	0.38	1	0.38	10.01	0.0035	significant
APP·MEL (APP-MEL)	0.40	1	0.40	10.73	0.0026	significant
Residual	1.17	31	0.04			
Lack of Fit	1.01	26	0.04	1.23	0.4504	not significant
Pure Error	0.16	5	0.03			
Cor Total	6.61	39				

**Table 5:** Fit statistics of the selected model, representing correlation factors R^2^, adjusted R^2^, and predicted R^2^.

Mean	5.28	R^2^	0.823
Standard deviation	0.194	Adjusted R^2^	0.777
CoV %	3.68	Predicted R^2^	0.651
		Adequate Precision	18.565

**Table 6:** Thermogravimetric analysis results of components: Mass loss rate peaks in normal atmosphere and remaining residue. Temperatures represent means of each decomposition step.

	Epoxy	APP	ATH	EG	MEL	CF
1st Peak in MLR (Temp./mass loss)	366 °C/70.9%	335 °C/17.7%	290 °C/29.2%	245 °C/16.2%	340 °C/100%	761 °C/95.6%
2nd Peak in MLR (Temp./mass loss)	535 °C/28.8%	624 °C/58.9%	-	794 °C/80.0%	-	-
Residue (weight)	0.1%	17.4%	65.5%	3.8%	0.0%	0.7%

**Table 7:** Thermogravimetric analysis results of mixtures and composites: Mass loss rate peaks in normal atmosphere and remaining residue. Temperatures represent means of each decomposition step.

Peak in MLR	EP CF	8	8 CF	13	13 CF	29	29 CF	33	33 CF
1st Peak (Temp./mass loss)	363 °C 39.3%	325 °C 32.4%	324 °C 23.4%	301 °C 14.1%	300 °C 10.1%	337 °C 47.5%	338 °C 24.7%	297 °C 23.7%	278 °C 12.5%
2nd Peak (Temp./mass loss)	535 °C 15.9%	354 °C 17.1%	356 °C 4.6%	377 °C 41.6%	379 °C 23.4%	619 °C 46.8%	580 °C 19.0%	375 °C 28.0%	380 °C 16.5%
3rd Peak (Temp./mass loss)	759 °C 44.8%	422 °C 7.6%	525 °C 17.0%	519 °C 25.5%	512 °C 18.2%	n/a	885 °C 53.0%	519.5 °C 33.9%	519 °C 13.9%
4th Peak (Temp./mass loss)	n/a	638 °C 27.0%	860 °C 49.5%	768 °C 6.1%	778 °C 38.1%	n/a	n/a	n/a	761 °C 53.3%
Residue (weight)	0.0%	15.9%	5.9%	13.8%	6.9%	0.0%	0.0%	6.9%	3.2%

**Table 8:** Cone calorimetry test of selected polymer and CFRP mixtures. (a) and (b) represent singular results from the double determination.

	**Polymer Mixtures**									
	**Run No. 40**		**Run No. 8**		**Run No. 13**		**Run No. 29**		**Run No. 33**	
	**(a)**	**(b)**	**(a)**	**(b)**	**(a)**	**(b)**	**(a)**	**(b)**	**(a)**	**(b)**
THR_100s_ [MJ/m^2^]	65.5	60.5	12.0	11.9	20.6	16.3	14.2	13.2	18.3	18.0
THR_600s_ [MJ/m^2^]	114.3	98.6	72.3	77.5	100.7	102.6	62.8	61.3	80.9	85.4
Average HRR_180s_ $\left[\mathrm{kW}/m^{2}\right]$	339	332	96	99	168	166	107	100	139	153
pHRR $\left[\mathrm{kW}/m^{2}\right]$	659	571	164	165	249	252	141	129	210	224
Mass [g]	46.11	40.13	55.44	54.62	50.50	54.99	52.5	56.55	53.14	54.70
Spec. THR_600s_ [MJ/m^2^·g]	2.48	2.46	1.31	1.42	1.99	1.86	1.20	1.08	1.52	1.56
TTI [s]	26	32	32	32	37	43	35	35	39	40
pCOP [ppm]	536	523	199	197	143	130	197	173	152	138
pCO_2_P [ppm]	26,947	28,931	3326	3656	9500	8441	5810	6045	9730	9065
	**CFRP Mixtures**									
	**Run No. 40 CF**		**Run No. 8 CF**		**Run No. 13 CF**		**Run No. 29 CF**		**Run No. 33 CF**	
	**(a)**	**(b)**	**(a)**	**(b)**	**(a)**	**(b)**	**(a)**	**(b)**	**(a)**	**(b)**
THR_100s_ [MJ/m^2^]	27.1	24.6	11.5	14.6	11.8	10.7	12.2	15.7	12.65	13.6
THR_600s_ [MJ/m^2^]	95.0	111.0	30.0	35.6	34.1	24.7	21.9	21.7	33.0	41.9
Average HRR_180s_ [kW/m^2^]	289	273	95	97	80	69	76	97	91	98
pHRR [kW/m^2^]	537	416	153	135	110	99	139	191	155	134
Mass [g]	58.06	57.87	39.39	43.75	42.5	40.2	40.35	41.05	44.38	44.70
Spec. THR_600s_ [MJ/m^2^·g]	1.63	1.92	0.76	0.81	0.8	0.62	0.54	0.53	0.74	0.94
TTI [s]	38	37	28	26	29	28	27	22	27	24
pCOP [ppm]	151	181	102	143	53	88	99	135	73	56
pCO_2_P [ppm]	9506	11509	2285	2636	3474	3643	2809	3262	3283	3886
	**Mean (CoV) Differences between Polymer to CFRP Samples ^1^**									
∆Spec. THR_600s_	28.1% (31.0%)		42.5% (1.6%)		63.2% (7.7%)		53.0% (5.4%)		45.5% (18%)	
∆Avg. HHR_180s_	16.3% (13.1%)		1.5% (45.2%)		55.4% (7.7%)		16.0% (114.9%)		35.2% (3.2%)	
∆pHHR	22.8% (26.7%)		12.4% (65.22%)		58.3% (5.9%)		−23.3% (150%)		33.2% (29.8%)	
∆TTI	−29.3% (8.3%)		15.6% (2.6%)		28.8% (6.5%)		30.0% (7.2%)		35.4% (5.1%)	
∆pCOP	68.6% (7.3%)		38.1% (12.2%)		48.4% (20.9%)		36.8% (15.5%)		55.5% (12.7%)	
∆pCO_2_P	62.4% (9.3%)		29.5% (8.4%)		60.3% (5.9%)		48.8% (6.7%)		61.9% (8.4%)	

^1^ Differences calculated on the basis of polymer mixtures. Positive values indicate decrease in carbon-fiber-containing mixtures.

## Data Availability

The data presented in this study are available on request from the corresponding authors. The data are not publicly available due to privacy restrictions.
